# Perceptions and knowledge of machine learning for paediatric related decision support in emergency care – A UK and Ireland network survey study of clinician leaders

**DOI:** 10.1371/journal.pdig.0001213

**Published:** 2026-02-09

**Authors:** Fiona Leonard, Mark D Lyttle, Dympna O’Sullivan, John Gilligan, Damian Roland, Michael Barrett, on behalf of PERUKI

**Affiliations:** 1 Department of Digital Health, Children’s Health Ireland, Dublin, Ireland; 2 School of Computer Science, Technological University Dublin, Dublin, Ireland; 3 Emergency Department, Bristol Royal Hospital for Children, Bristol, United Kingdom; 4 Research in Emergency Care Avon Collaborative Hub (REACH), University of the West of England, Bristol, United Kingdom; 5 Emergency Department, Perth Children’s Hospital, Perth, Australia; 6 Digital Futures Research Hub, Technological University Dublin, Dublin, Ireland; 7 Paediatric Emergency Medicine Leicester Academic (PEMLA) Group, Children’s Emergency Department, Leicester Royal Infirmary, Leicester, United Kingdom; 8 SAPPHIRE Group, Health Sciences, Leicester University, Leicester, United Kingdom; 9 Paediatric Emergency Research and Innovation (PERI), Department of Emergency Medicine, Children’s Health Ireland at Crumlin, Dublin, Ireland; 10 Women’s and Children’s Health, School of Medicine, University College Dublin, Dublin, Ireland; The University of Arizona, UNITED STATES OF AMERICA

## Abstract

This study explores clinician leaders understanding and perception at site level towards machine learning (ML) decision support tools for paediatric related emergency care across the UK and Ireland, essential in guiding safe and effective frontline implementation. A cross-sectional online survey was distributed via Paediatric Emergency Research United Kingdom and Ireland (PERUKI) to the lead for digital systems or PERUKI site lead, with one response sought per site. Survey development was in REDCap, and descriptive analysis (counts, percentages) was primarily performed. The response rate was 86.7% (65/75), mostly from England (83.1%). While 80.0% understood ‘Artificial Intelligence’, fewer understood advanced concepts such as ‘Deep Learning’ (32.3%). Most clinicians believed ML will support decision making (83.1%), would be willing to use (87.7%), and the future of decision making is a combination of human and ML (83.1%). Barriers included concerns about bias (61.5%), ML accuracy (56.9%), and inadequate information technology infrastructure (67.7%). Digital leads were more concerned about ML accuracy than non-digital (68.2% vs. 51.2%). Among potential applications, antimicrobial stewardship ranked highest (90.8%), and diagnosis of mental health conditions lowest (24.6%). Strong interest in ML tools for decision support in paediatric emergency care was evident, though concerns about bias, accuracy, and infrastructure must be addressed. Ongoing co-design with clinicians is critical in ensuring these tools are trusted, useful and suited to paediatric emergency care. Targeted education, digital leadership, and strategic investment in infrastructure and governance are essential for the successful adoption and integration of ML in clinical workflows.

## Author summary

Within emergency care we are seeing a rapid growth in the research, development and frontline use of machine learning based tools for decision support, yet very little is known about the intended users understanding, opinions, experience of this technology and supporting structures. With any new technology a greater understanding leads to better adoption and the clinicians who will use these tools should be directly involved in their design, implementation and evaluation to ensure that these tools are clinically relevant and usable in practice. Through our survey of clinical site leads (key drivers, influencing the adoption of ever advancing technology), the findings provide critical insight into what clinicians are most concerned about, their perceptions, understanding, what applications they view as most clinically relevant and their willingness to be involved in future research and development of these tools. The results also revealed many important themes such as infrastructure readiness, trust, explainability, clinical integration, targeted education and human-artificial intelligence collaboration. These findings will contribute to shaping the future of research, development, education, governance and policy within this rapidly growing area.

## Introduction

Despite increasing research on machine learning (ML) for clinical decision support, its understanding, adoption, use, and perception among emergency department (ED) clinicians remain underexplored. The rapid growth of ML, fuelled by electronic health records (EHRs) [1], highlights the need for further study in ED settings. The potential application of these tools are numerous, supporting decision making from when the patient arrives in the ED, during that visit and through to discharge [2] (e.g. quickly predicting triage, risk stratifying the most unwell patients [3], artificial intelligence (AI) assisted radiology diagnosis [4], and predicting discharge outcomes for bed planning [5, 6]). However, obstacles to adopting into real-world practice are abundant, with concerns including explainability, data bias, socio-technical, ethical, workforce, liability, patient safety, and the ability to integrate into clinical workflows [7–9]. The role of the institutional and departmental digital leads is integral to the adoption of these technologies.

Digital leaders take a more proactive role in advancing their organisations, supporting the adoption of modern and ever evolving digital healthcare technologies [10]. Digital leadership can positively transform attitudes and anxiety towards AI [11], therefore this role is pivotal in fostering greater acceptance, confidence, and engagement in the integration and use of ML decision support tools. The digital lead in this study refers to a clinician (typically a consultant) who takes on digital responsibilities in addition to their clinical duties within their department. The training/technical skills of this group are undetermined.

While the development of ML-based decision support tools in emergency care is expanding, prior research points to a lack of focus on paediatric-specific applications [12]. This study sought to assess the knowledge, perceptions, and obstacles to the adoption of ML based decision support tools among digital (if the position existed) and non-digital clinical leaders in paediatric emergency care across the United Kingdom and Ireland. Through surveying both digital and non-digital leaders, the study aimed to capture perspectives on current and potential use of ML in clinical settings, key challenges, and the crucial role of digital leadership which is instrumental in driving AI and ML adoption.

### Key concepts

- **Artificial Intelligence:** The ability of computers or machines to mimic intelligent behaviour, generating results that were once believed to require human intelligence [13].
- **Machine Learning:** A sub field of Artificial Intelligence. The ability of computers to learn and adapt on their own by analysing patterns in data using algorithms and statistical models, without needing explicit instructions [14].
- **Knowledge Based Systems:** These systems follow explicit instructions, typically structured as IF-THEN rules, where data is retrieved and assessed to determine the appropriate output or action [15].
- **Non-knowledge Based Systems:** These systems leverage Artificial Intelligence, Machine Learning or statistical models trained on datasets enabling computers to learn from past experiences and find patterns in data to provide outcomes, rather than being explicitly programmed [15].
- **Digital Maturity:** Digital maturity in healthcare describes the degree to which digital technologies are utilised and embedded to enable the provision of high-quality health services [16].
- **Artificial Intelligence Maturity:** How advanced an organisation is in implementing artificial intelligence, reflected in enhanced organisational capabilities and return on investment [17].

## Materials and methods

A cross-sectional online and anonymised survey study was conducted. Two separate surveys were developed as part of a broader study. This paper reports on the first survey, which targeted clinician site leaders across the UK and Ireland. A second survey, aimed at capturing the knowledge and views of the wider ED workforce (including frontline doctors, nurses, and ED support staff), was also conducted and will be reported and published separately. The survey questions were primarily informed by previous research [18–23] and refined through input, discussion and consensus among the study investigators (survey sections in Table 1), several of whom are extensively experienced in survey design. Survey usability and technical testing was undertaken by the study investigators and an independent emergency clinician reviewer, with revisions made based on feedback received. The study proposal and survey questions were initially reviewed by the Paediatric Emergency Research in the United Kingdom and Ireland (PERUKI) governance and research steering committees, whose feedback from 21 independent emergency medicine clinician respondents resulted in refinements to the study design, survey questions and the development of a video explaining key AI concepts to help respondents understand questions being asked in the survey. The survey questions are detailed in S1 File.

**Table 1:** Survey sections, with number of questions and answer types.

Survey Section	Number of questions	Types
Study participant information	6	Multiple choice, single answer
Site and participant demographics	5-9	Multiple choice, drop-down list Text Multiple choice, single answer Multiple choice, multiple answer
Confidence in understanding key artificial intelligence concepts	6-11	Likert (5 values) Text Multiple choice, single answer
Perceptions, concerns, and the future of machine learning for decision support	16	Likert (5 values)
Current information technology set up and current experience with clinical decision support system tools	9-18	Multiple choice, single answer Text Multiple choice, multiple answer
Reasons machine learning decision support tools are not used in their clinical setting	0-10	Likert (5 values) Text
Training in machine learning	3-4	Multiple choice, single answer Text Multiple choice, multiple answer
Areas of application for machine learning in emergency medicine	24-25	Likert (5 values) Text
Data repository with anonymised patient data for research purposes	5	Likert (5 values) Multiple choice, single answer

The online survey was developed in Research Electronic Data Capture (REDCap) [24,25], a secure online software platform designed to facilitate data collection for research. Branching logic was used on some questions which varied the number of questions asked depending on the responses provided. For sections with many questions, random order was utilised to reduce study fatigue and enhance the viability of the responses. All except two open questions (branching logic dependent) were mandatory. Anonymised data was stored securely on a server in Technological University Dublin (TU Dublin), accessible to the study investigators only.

An educational video (3.5 minutes) was embedded to explain key decision support types and AI concepts (https://vimeo.com/892770833/180bc711e1?). Participants were initially asked five questions to assess their understanding of key AI concepts before watching the video. After viewing the video, they were asked if they were satisfied with their initial understanding. Among those who responded negatively, the five questions were repeated.

The study was delivered across PERUKI. Sites comprise a range of types and locations, including stand-alone paediatric and mixed adult/paediatric ED, in urban and rural settings. The survey link was distributed to one person at each of the 75 PERUKI sites; where a digital lead existed, they were the recipient, and where there was no digital lead, the response was provided by the PERUKI site lead. One response per site was sought, consistent with standard methodology used in many previous PERUKI surveys [26,27], where a designated lead clinician provides a site-level response to represent high level departmental practice or perspectives. The recipients were all clinical leaders, predominately consultant doctors. The duration of the survey was four weeks beginning on 14^th^ June 2024 and ending on 12^th^ July 2024. Three reminders were sent, two weeks and three weeks after opening and 48 hours before the close of the survey.

Data exported from REDCap was prepared for analysis by checking and adjusting formats, removing incomplete surveys, adding summary columns, and verifying data integrity in both REDCap and Microsoft Excel. Descriptive analysis, including counts, percentages, and chart visualisation, was conducted in Microsoft Excel. Where relevant, figures and tables were stratified by group (digital, non-digital, and all respondents) to illustrate response patterns across these subgroups. Percentages shown in the results for Likert scales, summed ‘Agree’ and ‘Strongly Agree’ together, ‘Disagree’ and ‘Strongly Disagree’ and likewise summing ‘Likely’ and ‘Extremely Likely’ where applicable.

Inferential analyses were conducted to compare responses between digital and non-digital leads across several domains, including knowledge of key AI concepts, perceptions, concerns, future, potential applications of ML, barriers to implementation, and views on data sharing. Comparisons were also made across groups based on years of clinical experience, limited to knowledge of AI concepts and perceptions, concerns and future of ML. Categorical variables (Likert responses dichotomised as ‘Agree’ v ‘Other’ and ‘Likely’ v’Other’) were analysed using Chi-squared tests with Yates continuity correction or Fishers exact tests, depending on expected cell counts. Where appropriate, odds ratios and confidence intervals were calculated. A p-value < 0.05 was considered statistically significant. All inferential analyses were carried out using R (version 4.3.3). The study adhered to the Checklist for Reporting Results of Internet E-Surveys (CHERRIES) guidelines [28], with the completed checklist provided in S2 File.

### Ethical approval and participant consent

Ethical approval for this survey was received from both Children’s Health Ireland (REC-281–23) and TU Dublin (LEWS-023–83) Research Ethic Committees. Prior to gaining access to the survey, participants were required to provide electronic (written) informed consent by affirming six mandatory statements regarding their understanding of the study, the terms and their agreement to participate (S1 File).

## Results

Only completed responses were included in the results. The survey response rate was 86.7% (65/75 sites) after thirteen incomplete or duplicate responses were excluded. Most sites were in England (83.1%, 54/65) and treated both paediatric and adult patients (40/65, 61.5%). The digital lead position existed in 60.0% (39/65) of departments, and 83.1% (54/65) utilised healthcare records to drive improvements. Patient records were predominantly recorded electronically (Table 2).

**Table 2:** Site Characteristics.

Characteristic	Value (n = 65) n (%)
**Country/Constituent Country**	
England	54 (83.1)
Republic of Ireland	6 (9.2)
Scotland	3 (4.6)
Wales	1 (1.5)
Northern Ireland	1 (1.5)
**Site Type**	
Paediatric and Adult	40 (61.5)
Paediatrics	25 (38.5)
**Does a digital lead position exist in your department?**	
Yes	39 (60.0)
No	26 (40.0)
**Does your department utilise data from healthcare records to drive improvements?**	
Yes	54 (83.1)
No	11 (16.9)
**Emergency department patient records are recorded on**	
Fully electronic	33 (50.8)
Paper and electronic system	29 (44.6)
Paper based	3 (4.6)
**Type of Information System**	
Emergency Department module within a hospital wide EHR System	28 (43.1)
Integrated Emergency Department Information System	20 (30.8)
Standalone Emergency Department Information System	10 (15.4)
Hybrid System	7 (10.8)

The role of digital lead was held by 33.8% (22/65) respondents; however, 17/65 respondents completed the survey in place of the digital lead. In free-text responses, the respondents explained that this was due to the absence of a designated paediatric emergency medicine (PEM) digital lead, the role being covered by adult ED lead, paediatric or non-clinical leads. Some respondents reported that this role was temporarily vacant or shared among many team members.

Respondents were predominantly consultants, representing 95.5% (21/22) of digital leads and 97.7% (42/43) of non-digital leads. The majority had at least six years of experience in emergency medicine (digital leads: 77.3% (17/22), non-digital leads: 88.4% (38/43)). Most respondents (40.0%, 26/65) had paediatrics with PEM subspecialty training (Table 3).

**Table 3:** Respondent Characteristics.

Characteristic	All Positions (n = 65) n (%)	Digital (n = 22) n (%)	Non-digital (n = 43) n (%)
**Years Experience**			
0-2	1 (1.5)		1 (2.3)
3-5	9 (13.8)	5 (22.7)	4 (9.3)
6-10	17 (26.2)	5 (22.7)	12 (27.9)
10+	38 (58.5)	12 (54.5)	26 (60.5)
**Role**			
Consultant	63 (96.9)	21 (95.5)	42 (97.7)
Consultant Nurse	1 (1.5)		1 (2.3)
Paediatric Registrar	1 (1.5)	1 (4.5)	
**Clinician Type**			
Paediatrics with PEM subspecialty training	26 (40.0)	9 (40.9)	17 (39.5)
Emergency Medicine	17 (26.2)	7 (31.8)	10 (23.3)
Emergency Medicine with PEM subspecialty training	14 (21.5)	3 (13.6)	11 (25.6)
Paediatrics	6 (9.2)	2 (9.1)	4 (9.3)
Unknown	2 (3.1)	1 (4.5)	1 (2.3)

**Fig 1:**
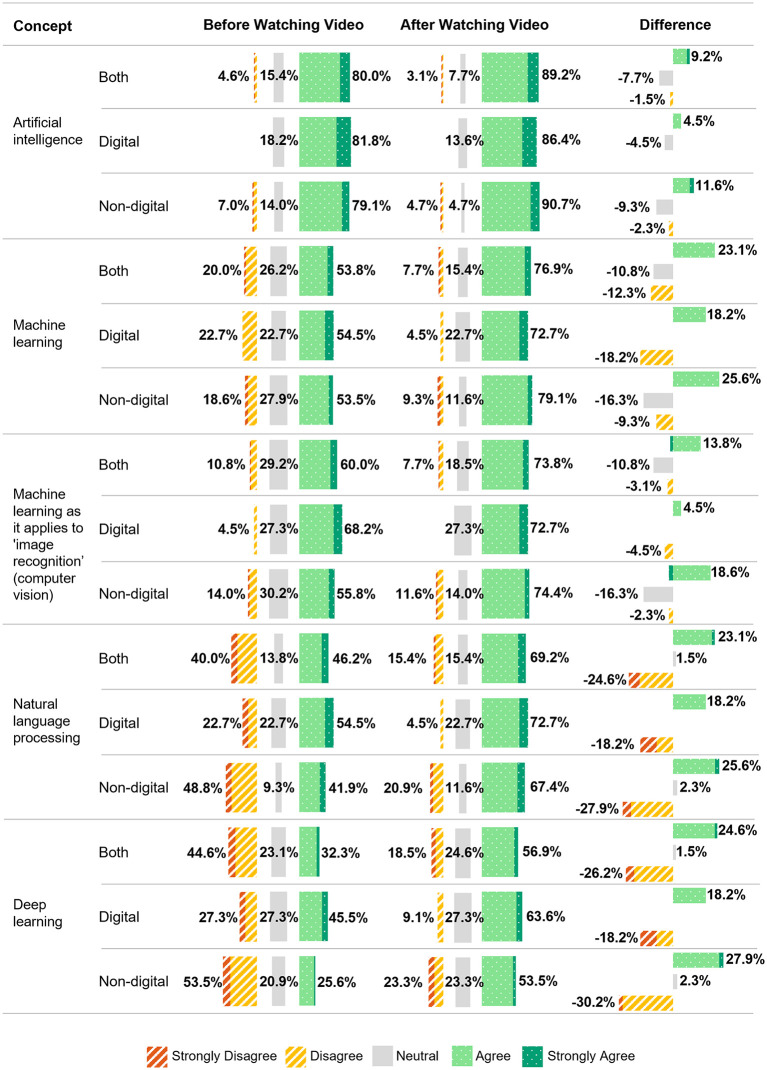
Confidence in Understanding Key Artificial Intelligence Concepts by digital and non-digital lead group, pre and post watching educational video.

After the video explaining AI concepts, 41.5% (27/65) revised their answers (digital leads: 31.8% (7/22), non-digital leads: 46.5% (20/43)). Digital leads understood all key concepts better pre-video, but post-video, non-digital leads outperformed them in 3/5 concepts. The highest level of understanding pre-video was for the concept of ‘Artificial Intelligence’ at 80.0% (52/65), which increased post-video to 89.2% (58/65). Confidence in deep learning rose for both groups, from 32.3% (21/65) to 56.9% (37/65), with non-digital leads seeing the biggest gain (+27.9% vs. + 18.2% for digital leads). Similarly, natural language processing understanding improved from 46.2% (30/65) to 69.2% (45/65), with non-digital leads again showing greater understanding (+25.6% vs. + 18.2%) (Fig 1).

Knowledge-based (rule based) decision support tools are more widely used than non-knowledge-based (ML) tools (38.5% (25/65) compared to 10.8% (7/65)). Among digital leads, 45.5% (10/22) used knowledge-based tools, compared to 34.9% (15/43) of non-digital leads. Only 10.8% (7/65) reported using non-knowledge-based tools (Table 4). When asked if they contributed to a project that uses ML in emergency medicine, 12.3% (8/65) confirmed that they had and 5/8 were digital leads.

**Table 4:** Use of knowledge and non-knowledge based tools.

	Knowledge based (Rule based)			Non-knowledge based (Machine Learning)		
	Digital (n = 22) n (%)	Non-digital (n = 43) n (%)	Both (n = 65) n (%)	Digital (n = 22) n (%)	Non-digital (n = 43) n (%)	Both (n = 65) n (%)
Yes	10 (45.5)	15 (34.9)	25 (38.5)	4 (18.2)	3 (7.0)	7 (10.8)
No	10 (45.5)	22 (51.2)	32 (49.2)	18 (81.8)	40 (93.0)	58 (89.2)
Unsure	2 (9.1)	6 (14.0)	8 (12.3)			

**Fig 2:**
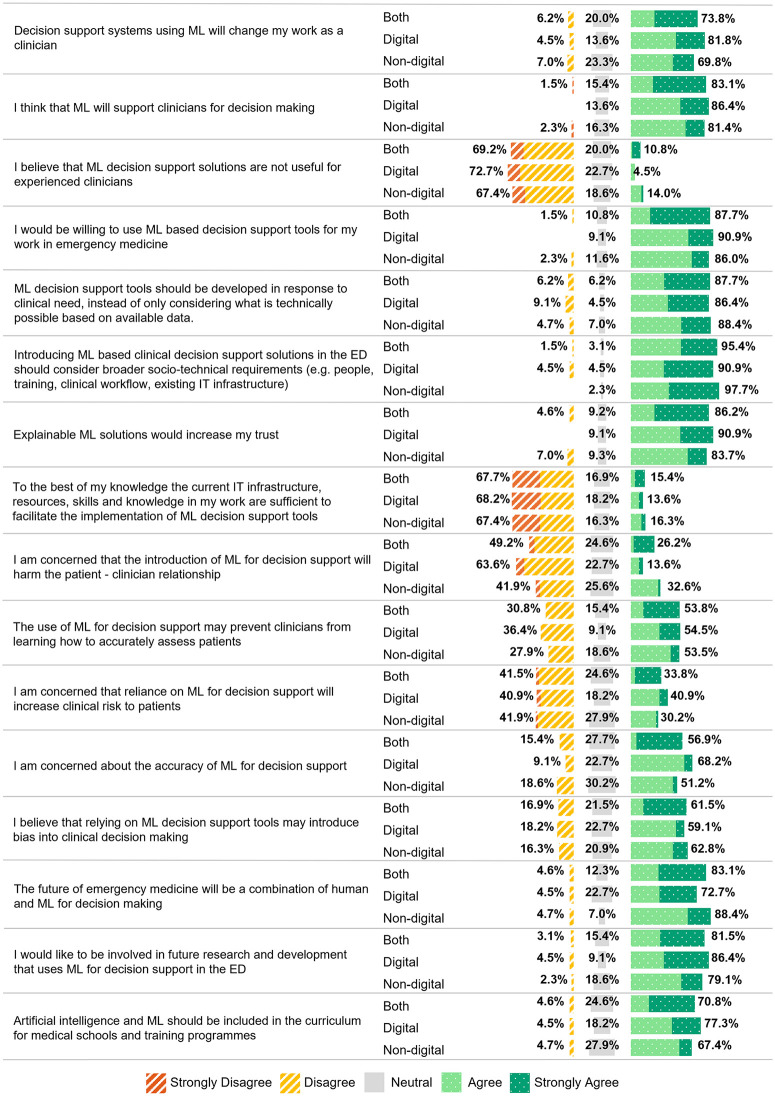
Perception, concerns, and the future of machine learning for decision support by digital and non-digital lead group. Abbreviations: ML, Machine Learning; ED, Emergency Department; IT, Information Technology.

Most respondents expressed willingness to use ML decision support tools (87.7%, 57/65) and 83.1% (54/65) believed these tools would support decision making. The majority agreeing these tools should be developed in response to clinical need (87.7%, 57/65) and should consider the broader socio-technical requirements (95.4%, 62/65). There was strong agreement that trust in these tools is underpinned by explainability (86.2%, 56/65). However, 67.7% (44/65) had significant concerns around infrastructure readiness and skilled resources, 56.9% (37/65) were concerned about ML accuracy and 53.8% (35/65) believed these tools may impact the ability for clinicians to accurately assess patients. Opinions on clinical risks and biases were divided, with 40.9% (9/22) of digital leads and 30.2% (13/43) of non-digital leads expressing concerns about increased risks and 59.1% (13/22) of digital leads and 62.8% (27/43) of non-digital leads raising concerns about potential biases. Overall, clinicians support integrating ML into emergency medicine, agreeing the future will be a combined human-ML approach (83.1%, 54/65). Non-digital leads (88.4%, 38/43) were more optimistic than digital leads (72.7%, 16/22), and a large proportion advocated its inclusion in medical training curricula (digital leads: 77.3% (17/22), non-digital leads: 67.4% (29/43)). Most (81.5%, 53/65) expressed interest in contributing to future research and development in ML decision support (Fig 2).

**Fig 3:**
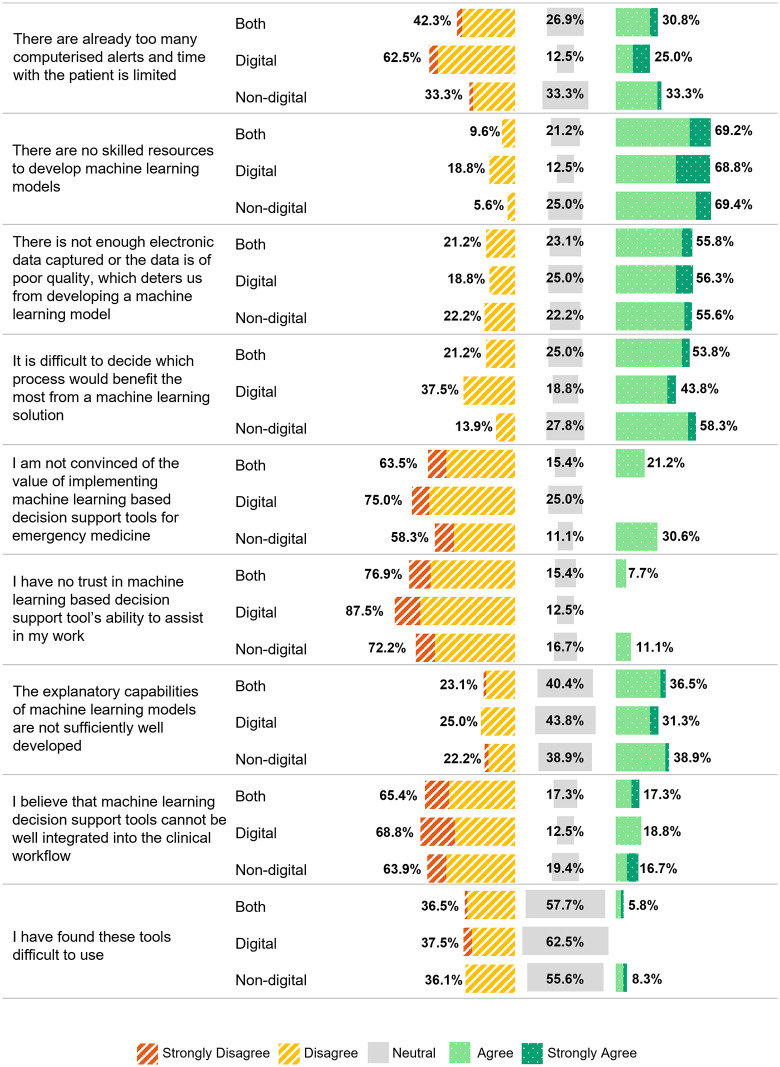
Opinion on why machine learning decision support tools are not used in the respondents clinical setting by digital and non-digital lead group.

Fifty two respondents (80.0%) indicated that ML tools were not currently implemented, and were subsequently presented with statements exploring potential reasons. The majority (69.2%, 36/52) agreed that a lack of skilled resources is a significant issue. Concerns about insufficient or poor-quality electronic data were also common, with 55.8% (29/52) agreeing that this is a deterrent. Difficulty in deciding which processes would benefit most from ML solutions was a strong factor (digital leads: 43.8% (7/16), non-digital leads: 58.3% (21/36)). Digital leads were more convinced of the value of ML tools for emergency medicine (75.0%, 12/16), whilst 30.6% (11/36) of non-digital leads were sceptical about their value. Lack of trust was higher among non-digital leads (11.1%, 4/36) compared to digital leads (0%, 0/16). ML models’ explanatory capabilities were considered underdeveloped (36.5%, 19/52), but most (65.4%, 34/52) disagreed that ML tools are too difficult to integrate into clinical workflows (Fig 3).

**Fig 4:**
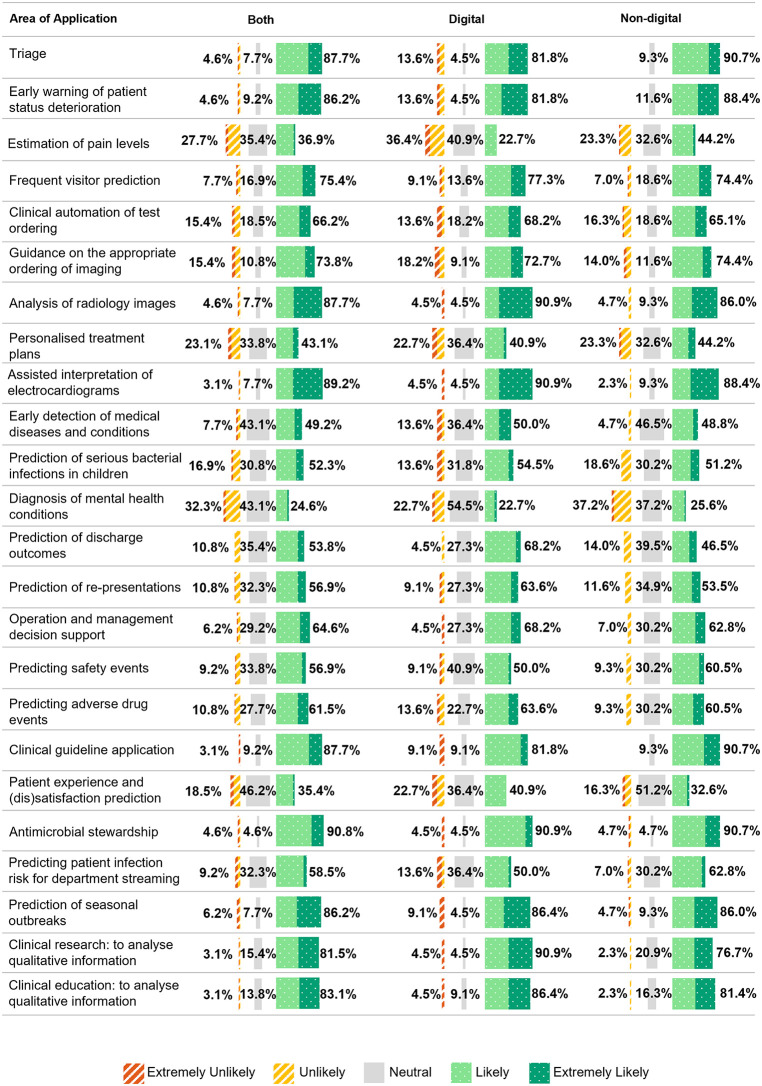
Areas of application of machine learning for decision support by digital and non-digital lead group.

The highest-rated applications of ML for decision support across both groups were antimicrobial stewardship (90.8%, 59/65), ML-assisted interpretation of electrocardiograms (ECG) (89.2%, 58/65), and analysis of radiology images (87.7%, 57/65). Lower-rated areas included diagnosis of mental health conditions (24.6%, 16/65) and patient experience and (dis)satisfaction prediction (35.4%, 23/65). (Fig 4).

Three respondents in the digital lead group (4.6%) confirmed that they had training in ML. One had accredited training and the other two were non-accredited or casual training. Most (89.2%, 58/65) expressed an interest in furthering their knowledge in ML.

**Fig 5:**
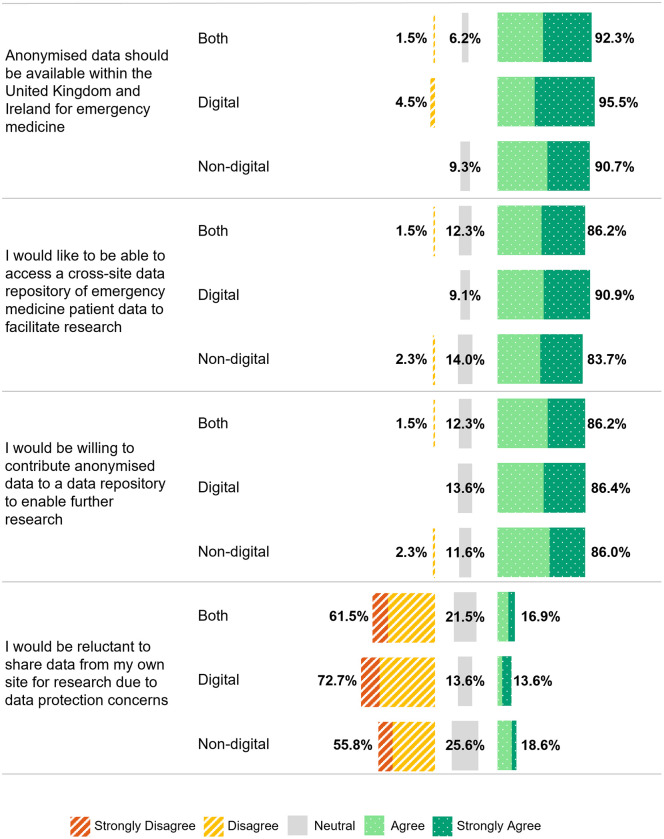
Assertions in relation to the sharing of fully anonymised patient data for research purposes by digital and non-digital lead group.

There was a strong consensus among the respondents, particularly digital leads, on the value of sharing anonymised patient data for research (digital leads: 95.5% (21/22), non-digital leads: 90.7% (39/43)). Most (86.2%, 56/65) expressed interest in both accessing and contributing data to a cross-site data repository to facilitate research. Concerns about data protection were lower among digital leads, with 13.6% (3/22) expressing reluctance to share data, compared to 18.6% (8/43) of the non-digital leads (Fig 5). When asked about how much of their emergency medicine data would be accessible for research purposes with the appropriate approvals in place, 44.6% (29/65) indicated that all or most of their data would be accessible, 18/65 reported that some would be accessible, 17/65 were unsure and one reported that none would be accessible.

Comparisons across digital vs non-digital leads and years of clinical experience revealed no statistically significant differences across the items tested in our survey. The full inferential results by subgroup and topic area are available in S3 File.

## Discussion

With the rapid growth in AI research within healthcare and the advent of advanced AI applications like ChatGPT [29], this survey comes at a pivotal time. To our knowledge, this is the first in children’s emergency care providing insights from frontline clinicians (specifically PERUKI site and digital leaders). A high response rate supports representation of a wide range of ED settings across the UK and Ireland. We have demonstrated both promise and challenges associated with the adoption of ML decision support tools in paediatric emergency care. While there is strong interest and willingness among clinicians to adopt these tools, gaps in AI literacy, infrastructure limitations, model accuracy, and concerns about bias present significant barriers. The educational video effectively reduced knowledge gaps, suggesting targeted training can improve clinician’s confidence in understanding key AI concepts, an essential step in adopting ML decision support tools. Digital leads were present across sites, but no significant differences in AI-related responses were found between digital and non-digital leads. ML tools were perceived to be most beneficial when based on clinical need. While many respondents reported that all or most of their data would be accessible for research with appropriate approvals, the variation in responses (including uncertainty and limited accessibility in some cases), highlights both opportunities and challenges in building cross-site research frameworks, provided concerns around data protection and security are addressed. These insights highlight the need for strategic investments in training, infrastructure, and leadership to ensure safe and equitable deployment of ML tools in clinical practice.

### Current knowledge, education and training

AI literacy involves understanding the fundamental functions of AI and how to utilise these applications effectively and responsibly [30]. The focus for most medical professionals is therefore about interacting with and using AI in healthcare rather than developing AI technologies or conducting AI research [31]. Understanding basic AI concepts is a key first step, as AI illiteracy can be a significant obstacle to adoption [32], especially for key decision makers. In this study, digital leads had a stronger understanding of all AI concepts before watching the video, likely due to greater exposure to digital systems. The video, which was designed to provide a brief baseline understanding of key concepts, helped narrow the gap in self-reported AI knowledge between groups, with non-digital leads showing greater improvement in understanding advanced concepts like natural language processing and deep learning. These findings align with a 2023 systematic review, were four out of 10 studies found that healthcare professionals and students reported low baseline AI knowledge and literacy. Across all included studies, AI training courses and their application in healthcare were regarded as essential for both professionals and students, with efforts focused on enhancing the educational infrastructure to support this need [33]. This highlights the need to integrate targeted AI education into professional development, ensuring all clinical leaders, regardless of prior experience, are equipped to make informed decisions about integrating ML in healthcare for decision support.

Scott et al [34] advise that an important strategic enabler for adoption is enhancing clinician AI literacy, including AI/ML tool design and evaluation to assess suitability and appropriate use. Most respondents valued education and training, expressing interest in furthering their knowledge (89.2%), and supporting its inclusion in medical school curricula and training programmes (70.8%). Researchers believe that medical education needs to expand beyond the foundational biology, clinical skills and new diagnostic and therapeutic trends, with a key focus on preparing students to thrive in a healthcare system increasingly influenced by advancements in AI [35]. According to Ng et al [30] and also inspired by Bloom taxonomy, AI literacy should include fundamental competencies such as knowledge and understanding, usage and application, development and evaluation, along with social responsibility and ethical awareness. Most respondents (81.5%) would like to be involved in future research and development of ML based decision support tools. This co-design is essential for clinical utility, and education in AI/ML can enhance collaboration between clinicians and data scientists. However, the knowledge gap between clinicians, engineers, and scientists continues to grow as technology in healthcare advances, leaving physicians ill-prepared to work with AI tools [36] and impacting future collaboration. Medical schools should explore ways of introducing ML training modules to understand the promise and potential pitfalls [37].

### Opportunities and challenges

Most respondents (83.1%) believed these tools would enhance clinical decision making, with 87.7% willing to adopt them. However, concerns remain, the greatest relating to explainable AI (XAI), with 86.2% agreeing this would increase trust. XAI methods provide the ability for human experts to understand explanatory factors behind AI decision making. These methods help identify which input features most influence a model’s output using established techniques such as Shapley values, Local Interpretable Model Agnostic Explanations, and integrated gradients [38]. However, true understanding requires not just technical explanations, but also the ability of humans to comprehend them. Achieving this requires new human-AI interfaces enabling domain experts to ask questions and interactively explore explanations [39]. These challenges highlight the importance of AI literacy, ensuring clinicians are equipped to interpret and critically engage with XAI outputs. Another significant concern was bias being introduced into clinical decision making (61.5%). Bias in healthcare may arise from systemic inequalities embedded in healthcare data [40], but occurs in most stages of ML development: formulation of the research problem, data collection, data pre-processing, model development and validation and model implementation [41], leading to inequitable decision making, potentially impacting treatments and outcomes. A set of principles: findable, accessible, interoperable and reusable (FAIR) that were originally intended to be applied to data are now being designed for use with AI models [42] to address bias. Removing bias requires consideration of several areas, within model design, training data, and interactions with both clinicians and patients [43].

Accuracy of these tools and clinical risk to patients were additional concerns. ML tools deployed in production must go through rigorous validation and have a proven level of accuracy that complies with clinical safety standards. Labkoff et al [44] through engagement with many diverse stakeholders produced guidelines for the development, validation and deployment of safe and trustworthy AI systems in healthcare. As patterns in data change over time, continuous monitoring and improvement of these models is important in the model lifecycle to detect performance degradation. With the European Union (EU) AI Act, high risk AI systems are required to set up post-market monitoring systems that will address any risks emerging from these systems to be addressed in a timely manner and as a legal requirement to report any breaches [45].

Many respondents (53.8%) believed using ML tools may prevent clinicians from learning how to accurately diagnose patients. It has been argued that incorporating ML decision support tools in medicine may lead to overreliance on automation, resulting in deskilling/loss of skills and curbing clinicians’ ability to learn, with reduced ability to make informed decisions when technology breaks down or fails [46]. However, this deskilling can be a result of clinicians’ own choices and external pressures of their work environment [47]. These tools are intended to support, not replace clinical judgement. The EU AI Act emphasises human oversight throughout the process for high risk AI systems and it is essential to ensure clinicians remain central in decision-making, understanding the limitations of the system, either disregarding or reversing outputs, and safely intervening and disabling the system when necessary [45,48].

Lastly, 67.7% believed current IT infrastructure, resources, skills, and knowledge were insufficient. Digital maturity levels across sites varied, with only half having fully electronic systems. Widespread implementation of AI requires significant IT system reconfiguration and supporting resources, which can be challenging in terms of securing investment amid competing healthcare priorities [7]. Interestingly, AI maturity models are now emerging to support the digital and AI maturity roadmap. These maturity models are useful tools in defining what degree of readiness an organisation is at in order to take advantage of AI [49].

### Potential applications

Among potential ML applications, antimicrobial stewardship, which focuses on appropriate antibiotic use was ranked one of the highest by both digital and non-digital leads at 90.8%. Antibiotics are often overprescribed in paediatric emergency medicine, partly due to clinician-caregiver interactions and diagnostic uncertainty, leading to risks such as antimicrobial resistance, adverse drug events and an increase in healthcare costs [50]. ML tools have the potential to reduce time spent reviewing data by anti-microbial team members, making predictions in seconds, freeing up time to focus more on diagnostic and therapeutic decisions instead [51]. In fact, the World Health Organization highlighted the importance of these programmes by publishing a practical toolkit in 2019 for low and middle income countries [52]. The paediatric emergency medicine priority setting partnership between PERUKI and the James Lind Alliance also listed how to safely reduce antibiotics use for children in their research priority list across the UK and Ireland [53]. The second highest ranked application was ML assisted interpretation of ECGs (89.2%), these tools could improve clinician interpretation of ECGs, reduce errors and expedite patient care in EDs [54]. The application ranked lowest by both groups at 24.6% was ML assisted diagnosis of mental health conditions. The number of paediatric patients presenting to the ED with mental health conditions is increasing globally, further straining already limited ED resources [55]. Shatt et al [56] carried out a scoping review of ML in mental health, with some promising applications that included detection and diagnosis of conditions such as depression, psychosis, schizophrenia and suicidal ideation, some of which included imaging data. However, diagnosis of these conditions may often take place in the community, outpatient or other specialised psychiatric settings rather than the ED. Clinicians in the ED are trained to assess and manage paediatric patients who present with mental health emergencies [57] as opposed to providing definitive diagnoses.

### Data sharing

The accuracy and generalisability of AI models are strongly influenced by the availability of large, diverse and representative training data reflecting the target population [58]. Sharing fully anonymised patient data to create a repository would greatly enhance research and innovation for ML decision support tool development across the UK and Ireland. Maassen et al [20] identified that 82.5% of surveyed German hospital physicians agreed on the value of making data available for research, compared to 92.3% from our results. Willingness to contribute anonymised data and access it was exceeded in our study, suggesting that efforts to develop a research data sharing framework would be broadly supported. Data protection concerns were evident, which also surfaced when asked how much data would be accessible, 27.7% reported that some would be accessible and 27.7% remained unsure or reported that none would be. This indicates a need for further reassurance and education on data security, in particular sophisticated techniques that can balance privacy protection with data utility [59], ensuring trust in data sharing. Federated learning, where ML models are trained on decentralised data, offers a promising alternative to centralised data sharing while addressing privacy concerns, however even this approach comes with challenges including data standardisation, potential model bias and high processing power requirements [44].

### Digital versus non-digital leads

Digital lead positions existed at 60% of the sites, indicating progress and investment in digital transformation. While inferential analyses revealed no statistically significant differences in responses between digital and non-digital leads, several descriptive trends were observed. Digital leads demonstrated a higher initial understanding of key AI concepts, greater confidence that ML will impact their clinical work, a desire to participate in research and development, and a stronger belief that AI and ML should be included in medical school curricula. They also exhibited fewer trust issues, recognised the value of implementing these tools for decision support, showed greater willingness to share data, and had fewer data protection concerns. In contrast, non-digital leads showed greater education benefits from the educational video, were more convinced that the future would be a human-ML collaboration for decision making, however expressed more concern about harm to the patient-clinician relationship, clinical value, trust, explanatory capabilities and potential bias. Some of these differences likely reflect the digital leads familiarity with the technology and its implementation. This highlights the need for broader AI education to bridge knowledge gaps, build trust, and foster collaboration in adopting AI tools in clinical practice for all clinicians. While our findings did not show statistically significant differences between digital and non-digital leads, the observed trends suggest that clinical digital leaders may play a valuable role in championing AI and ML adoption in clinical practice. Researchers found that digital transformation was accelerated with programmes such as the global digital exemplar programme which supports the mandatory introduction of clinical health informatics leaders locally such as a Chief Clinical Information Officer (CCIO) (a dual clinical-digital leadership role), which in turn has promoted the emergence of leadership positions such as a Chief Medical Information Officer and deputy CCIOs overseeing specific subdisciplines [60]. Embedding similar leadership roles at the departmental level can support organisational change by aligning clinical and digital priorities, building trust in new technologies, and engaging staff in the adoption process.

### Success factors for machine learning development

Success depends on selecting the right use cases with clear clinical value, Ramgopal et al [61] reports that AI clinical decision systems should answer clinically relevant questions. Engaging clinicians early in the research and development of AI tools ensures they are both practical and aligned with real-world clinical needs. In our study, only 12.3% (8/65) of respondents reported having previously contributed to a project using ML in emergency medicine, suggesting that current clinical involvement in ML development remains limited. This highlights an opportunity to increase interdisciplinary collaboration in future initiatives. Collaboration between clinicians, data scientists, and digital leaders is essential to the co-design process, as it fosters the development of more trustworthy and responsible XAI tools for high-stakes healthcare decisions [62], ultimately enhancing clinical adoption and improving patient outcomes. Increasing training and awareness is essential to bridge knowledge gaps, reduce resistance, and build trust in AI systems. Addressing common concerns such as explainability, bias, and patient safety is equally critical. Ensuring data quality and improving electronic health record infrastructure will enhance model performance. Finally, seamless clinical workflow integration is key to ensuring these tools support rather than disrupt clinical practice, enabling a more efficient human-ML partnership.

### Limitations

The survey was distributed through the PERUKI network, eliciting one response per site, reflecting the opinions of the leads as opposed to the wider ED workforce. Findings depended on the accurate self-reporting from a single respondent per site. Some respondents struggled with AI concepts that were explained in the educational video, evident from the low percentage of respondents who confirmed using knowledge (rule) based systems at 38.5%. Fewer digital leads compared to non-digital responded, potentially impacting on responses around AI readiness and infrastructure challenges. Inferential analyses were limited by sample size and group imbalance, particularly between digital and non-digital leads. This may have reduced the power to detect statistically significant differences, even where descriptive trends were observed. Finally, the variability in digital maturity across the sites would have influenced responses, however this was also an important insight.

## Conclusion

This study highlights a strong interest from clinician leaders to integrate ML decision support tools into clinical workflows, recognising the benefits to decision making. Challenges remain, including infrastructure readiness, explainability, trust, concerns about bias, potential clinician deskilling and how humans and AI can work effectively together, highlighting the need for careful implementation strategies and ongoing human-AI collaboration. While digital leads showed descriptively higher baseline understanding of key AI concepts, greater support for data sharing, and more confidence in the clinical value of ML tools, inferential analyses did not find statistically significant differences between groups. We demonstrated that educational interventions can enhance AI literacy. Support of varying degrees of data sharing presents important challenges and possibilities to create cross-site research frameworks to advance ML tool development. Maximising ML’s potential in decision support requires investment in training, infrastructure, and governance. Prioritising clinician engagement, responsible AI design, and seamless integration will ensure these tools enhance patient care. Further mixed methodology research (surveys, quantitative and qualitative research) should be carried out of the wider ED workforce and adult only EDs to ensure more inclusive and generalisable insights. As part of this broader study, we have conducted a follow-on survey of the wider ED workforce, and the results will be published separately.

## Supporting information

S1 File Survey Questionnaire. Details of study participant information and the survey questions.
(PDF)

S2 File Survey Checklist. Checklist for Reporting Results of Internet E-Surveys (CHERRIES).
(PDF)

S3 File Statistical Tests. Results of inferential statistical tests carried out.
(PDF)

S1 Acknowledgements Full list of acknowledged PERUKI member names. (PDF)
