# Supplementary material for: Perceptions and knowledge of machine learning for paediatric related decision support in emergency care – A UK and Ireland network survey study of clinician leaders
Source: PLOS Digit Health. 2026 Feb 9;5(2):e0001213. doi: 10.1371/journal.pdig.0001213 (PMC12885286; doi:10.1371/journal.pdig.0001213)
Supplement: S3 File — Results of inferential statistical tests carried out. (PDF) [file pdig.0001213.s003.pdf]

Supporting Information S3: Statistical Tests

Digital versus Non-Digital Leads

Table S3.1 Confidence in Understanding Key Artificial Intelligence Concepts – Pre video

Statement	Test	Digital Lead (Agree/Other)	Non-digital Lead (Agree/Other)	p-value	Odds ratio [95% CI]
I am confident that I understand the concept of artificial intelligence	Fisher's exact	18/4	34/9	1.00	1.19 [0.32-4.41]
I am confident that I understand the concept of machine learning	Chi-squared with Yates continuity	12/10	23/20	0.86	1.04 [0.37-2.93]
I am confident that I understand the concept of machine learning as it applies to 'image recognition' (computer vision)	Chi-squared with Yates continuity	15/7	24/29	0.49	1.70 [0.58-5.00]
I am confident that I understand the concept of natural language processing	Chi-squared with Yates continuity	12/10	18/25	0.48	1.67 [0.59-4.69]
I am confident that I understand the concept of deep learning	Chi-squared with Yates continuity	10/12	11/32	0.18	2.42 [0.82-7.16]

Table S3.2 Whether answers changed post video

Question	Test	Digital Lead (Yes/No)	Non-digital Lead (Yes/No)	p-value	Odds ratio [95% CI]
Having watched the video do you still stand by the original answers you supplied for Artificial Intelligence and Machine Learning concept understanding?	Chi-squared with Yates continuity	15/7	23/20	0.38	1.86 [0.63-5.48]

Table S3.3 Confidence in Understanding Key Artificial Intelligence Concepts - Post video (For those that changed their answers)

Statement	Test	Digital Lead (Agree/Other)	Non-digital Lead (Agree/Other)	p-value	Odds ratio [95% CI]
I am confident that I understand the concept of artificial intelligence	Fisher's exact	7/0	19/1	1.00	1.15 [0.04-31.59]
I am confident that I understand the concept of machine learning	Fisher's exact	5/2	19/1	0.16	0.13 [0.01-1.76]
I am confident that I understand the concept of machine learning as it applies to 'image recognition' (computer vision)	Fisher's exact	5/2	15/5	1.00	0.83 [0.12-5.72]
I am confident that I understand the concept of natural language processing	Fisher's exact	5/2	14/6	1.00	1.07 [0.16-7.15]
I am confident that I understand the concept of deep learning	Fisher's exact	4/3	12/8	1.00	0.89 [0.16-5.08]

Table S3.4 Perception, concerns, and the future of machine learning for decision support

Statement	Test	Digital Lead (Agree/Other)	Non-digital Lead (Agree/Other)	p-value	Odds ratio [95% CI]
Decision support systems using machine learning will change my work as a clinician	Chi-squared with Yates continuity	18/4	30/13	0.45	0.51 [0.15-1.82]
I think that machine learning will support clinicians for decision making	Fisher's exact	19/3	35/8	0.74	0.69 [0.16-2.92]
I believe that machine learning decision support solutions are not useful for experienced clinicians	Fisher's exact	1/21	6/37	0.41	3.41 [0.38-30.24]

Statement	Test	Digital Lead (Agree/Other)	Non-digital Lead (Agree/Other)	p-value	Odds ratio [95% CI]
I would be willing to use machine learning based decision support tools for my work in emergency medicine	Fisher's exact	20/2	37/6	0.71	0.62 [0.11-3.34]
Machine learning decision support tools should be developed in response to clinical need, instead of only considering what is technically possible based on available data	Fisher's exact	19/3	38/5	1.00	1.2 [0.26-5.56]
The introduction of machine learning based clinical decision support solutions in the ED should consider broader socio-technical requirements (e.g. people, training, clinical workflow, existing information technology infrastructure)	Fisher's exact	20/2	42/1	0.26	4.20 [0.36-49.10]
Explainable machine learning solutions would increase my trust	Fisher's exact	20/2	36/7	0.71	0.51 [0.10-2.72]
To the best of my knowledge the current information technology infrastructure, resources, skills and knowledge in my work are sufficient to facilitate the implementation of machine learning decision support tools	Fisher's exact	3/19	7/36	1.00	1.23 [0.29-5.32]
I am concerned that the introduction of machine learning for decision support will harm the patient - clinician relationship	Chi-squared with Yates continuity	3/19	14/29	0.18	3.06 [0.77-12.09]
The use of machine learning for decision support may prevent clinicians from learning how to accurately assess patients	Chi-squared with Yates continuity	12/10	23/20	0.86	0.96 [0.34-2.69]
I am concerned that reliance on machine learning for decision support will increase clinical risk to patients	Chi-squared with Yates continuity	9/13	13/30	0.56	0.63 [0.22-1.83]
I am concerned about the accuracy of machine learning for decision support	Chi-squared with Yates continuity	15/7	22/21	0.30	0.49 [0.17-1.44]
I believe that relying on machine learning decision support tools may introduce bias into clinical decision making	Chi-squared with Yates continuity	13/9	27/16	0.98	1.17 [0.41-3.34]
The future of emergency medicine will be a combination of human and machine learning for decision making	Fisher's exact	16/6	38/5	0.16	2.85 [0.76-10.70]
I would like to be involved in future research and development that uses machine learning for decision support in the Emergency Department	Fisher's exact	19/3	34/9	0.74	0.60 [0.14-2.47]
Artificial intelligence and machine learning should be included in the curriculum for medical schools and training programmes	Chi-squared with Yates continuity	17/5	29/14	0.59	0.61 [0.19-1.99]

Table S3.5 Opinion on why machine learning decision support tools are not used in the respondents clinical setting

Statement	Test	Digital Lead (Agree/Other)	Non-digital Lead (Agree/Other)	p-value	Odds ratio [95% CI]
There are already too many computerised alerts and time with the patient is limited	Fisher's exact	4/12	12/24	0.75	1.50 [0.40-5.65]
There are no skilled resources to develop machine learning models	Fisher's exact	11/5	25/11	1.00	1.03 [0.29-3.69]
There is not enough electronic data captured or the data is of poor quality, which deters us from developing a machine learning model	chi-squared with Yates continuity	9/7	20/16	0.80	0.97 [0.30-3.19]
It is difficult to decide which process would benefit the most from a machine learning solution	Chi-squared with Yates continuity	7/9	21/15	0.50	1.80 [0.55-5.91]
I am convinced of the value of implementing machine learning based decision support tools for emergency medicine	Chi-squared with Yates continuity	12/4	21/15	0.40	0.47 [0.13-1.73]
I have trust in machine learning based decision support tools ability to assist in my work	Fisher's exact	14/2	26/10	0.30	0.37 [0.07-1.94]
The explanatory capabilities of machine learning models are not sufficiently well developed	Chi-squared with Yates continuity	5/11	14/22	0.83	1.40 [0.40-4.89]
I believe that machine learning decision support tools cannot be well integrated into the clinical workflow	Fisher's exact	3/13	6/30	1.00	0.87 [0.19-4.01]
I have not found these tools difficult to use	Chi-squared with Yates continuity	6/10	13/23	0.83	0.94 [0.28-3.19]

Table S3.6 Assertions in relation to the sharing of fully anonymised patient data for research purposes

Statement	Test	Digital Lead (Agree/Other)	Non-digital Lead (Agree/Other)	p-value	Odds ratio [95% CI]
Anonymised data should be available within the United Kingdom and Ireland for emergency medicine	Fisher's exact	21/1	39/4	0.65	0.46 [0.05-4.43]
I would like to be able to access a cross-site data repository of emergency medicine patient data to facilitate research	Fisher's exact	20/2	36/7	0.71	0.51 [0.10-2.72]
I would be willing to contribute anonymised data to a data repository to enable further research	Fisher's exact	19/3	37/6	1.00	0.97 [0.22-4.33]
I would be reluctant to share data from my own site/research due to data protection concerns	Fisher's exact	3/19	8/35	0.74	1.45 [0.34-6.11]

Table S3.7 Areas of application of machine learning for decision support

Application	Test	Digital Lead (Likely/Other)	Non-digital Lead (Likely/Other)	p-value	Odds ratio [95% CI]
Triage	Fisher's exact	18/4	39/4	0.43	2.17 [0.49-9.65]
Early warning of patient status deterioration	Fisher's exact	18/4	38/5	0.47	1.69 [0.40-7.05]
Estimation of pain levels	Chi-squared with Yates continuity	5/17	19/24	0.15	2.69 [0.84-8.63]
Frequent visitor prediction	Chi-squared with Yates continuity	17/5	32/11	0.96	0.86 [0.26-2.87]
Clinical automation of test ordering	Chi-squared with Yates continuity	15/7	28/15	0.98	0.87 [0.29-2.60]
Guidance on the appropriate ordering of imaging	Chi-squared with Yates continuity	16/6	32/11	0.88	1.09 [0.34-3.49]
Analysis of radiology images	Fisher's exact	20/2	37/6	0.71	0.62 [0.11-3.34]
Personalised treatment plans	Chi-squared with Yates continuity	9/13	19/24	0.99	1.14 [0.40-3.24]
Machine learning assisted interpretation of electrocardiograms	Fisher's exact	20/2	38/5	1.00	0.76 [0.14-4.27]

Application	Test	Digital Lead (Likely/Other)	Non-digital Lead (Likely/Other)	p-value	Odds ratio [95% CI]
Early detection of medical diseases and conditions	Chi-squared with Yates continuity	11/11	21/22	0.86	0.96 [0.34-2.67]
Prediction model for serious bacterial infections in children	Chi-squared with Yates continuity	12/10	22/21	1.00	0.87 [0.31-2.45]
Diagnosis of mental health conditions	Chi-squared with Yates continuity	5/17	11/32	0.96	1.17 [0.35-3.92]
Prediction of discharge outcomes	Chi-squared with Yates continuity	15/7	20/23	0.16	0.41 [0.14-1.19]
Prediction of re-presentations	Chi-squared with Yates continuity	14/8	23/20	0.61	0.66 [0.23-1.89]
Operation and management decision support	Chi-squared with Yates continuity	15/7	27/16	0.88	0.79 [0.27-2.34]
Predicting safety events	Chi-squared with Yates continuity	11/11	26/17	0.59	1.53 [0.54-4.31]
Predicting adverse drug events	Chi-squared with Yates continuity	14/8	26/17	0.98	0.87 [0.30-2.53]
Clinical guideline application	Fisher's exact	18/4	39/4	0.43	2.17 [0.49-9.65]
Patient experience and (dis)satisfaction prediction	Chi-squared with Yates continuity	9/13	14/29	0.69	0.70 [0.24-2.02]
Antimicrobial stewardship	Fisher's exact	20/2	39/4	1.00	0.98 [0.16-5.79]
Predicting patient infection risk for department streaming	Chi-squared with Yates continuity	11/11	27/16	0.47	1.69 [0.60-4.77]
Prediction of seasonal outbreaks	Fisher's exact	19/3	37/6	1.00	0.97 [0.22-4.33]
Clinical research: to analyse qualitative information	Fisher's exact	20/2	33/10	0.20	0.33 [0.07-1.66]
Clinical education: to analyse qualitative information	Fisher's exact	19/3	35/8	0.74	0.69 [0.16-2.92]

Years of Experience

Table S3.8 Confidence in Understanding Key Artificial Intelligence Concepts – Pre video

Statement	Chi-square (χ^2)	df	p-value
I am confident that I understand the concept of artificial intelligence	1.32	3	0.72
I am confident that I understand the concept of machine learning	3.74	3	0.29
I am confident that I understand the concept of machine learning as it applies to 'image recognition' (computer vision)	3.20	3	0.36
I am confident that I understand the concept of natural language processing	0.90	3	0.83
I am confident that I understand the concept of deep learning	4.07	3	0.25

Table S3.9 Perception, concerns, and the future of machine learning for decision support

Statement	Chi-square (χ^2)	df	p-value
Decision support systems using machine learning will change my work as a clinician	6.26	3	0.10
I think that machine learning will support clinicians for decision making	1.20	3	0.75
I believe that machine learning decision support solutions are not useful for experienced clinicians	2.05	3	0.56
I would be willing to use machine learning based decision support tools for my work in emergency medicine	0.18	3	0.98
Machine learning decision support tools should be developed in response to clinical need, instead of only considering what is technically possible based on available data	1.08	3	0.78
The introduction of machine learning based clinical decision support solutions in the ED should consider broader socio-technical requirements (e.g. people, training, clinical workflow, existing information technology infrastructure)	0.58	3	0.90
Explainable machine learning solutions would increase my trust	4.36	3	0.23
To the best of my knowledge the current information technology infrastructure, resources, skills and knowledge in my work are sufficient to facilitate the implementation of machine learning decision support tools	0.38	3	0.94

Statement	Chi-square (χ^2)	df	p-value
I am concerned that the introduction of machine learning for decision support will harm the patient - clinician relationship	2.14	3	0.54
The use of machine learning for decision support may prevent clinicians from learning how to accurately assess patients	1.21	3	0.75
I am concerned that reliance on machine learning for decision support will increase clinical risk to patients	1.78	3	0.62
I am concerned about the accuracy of machine learning for decision support	1.74	3	0.63
I believe that relying on machine learning decision support tools may introduce bias into clinical decision making	7.58	3	0.06
The future of emergency medicine will be a combination of human and machine learning for decision making	2.50	3	0.47
I would like to be involved in future research and development that uses machine learning for decision support in the Emergency Department	0.84	3	0.84
Artificial intelligence and machine learning should be included in the curriculum for medical schools and training programmes	1.96	3	0.58
