# Supplementary material for: Perceptions and knowledge of machine learning for paediatric related decision support in emergency care – A UK and Ireland network survey study of clinician leaders
Source: PLOS Digit Health. 2026 Feb 9;5(2):e0001213. doi: 10.1371/journal.pdig.0001213 (PMC12885286; doi:10.1371/journal.pdig.0001213)
Supplement: S1 Acknowledgements — (PDF) [file pdig.0001213.s004.pdf]

Supporting Information S1 Acknowledgements

Acknowledged are the Paediatric Emergency Research United Kingdom and Ireland (PERUKI) site leads who distributed and for some sites also completed the survey:

Meriel Tolhurst-Cleaver, Alder Hey Children's Hospital NHS Foundation Trust, Liverpool;

Daniel Murrell, Bedfordshire Hospitals NHS Foundation Trust - Luton and Dunstable University Hospital;

Jonathan Adamson, Birmingham Children's Hospital; Charlotte Munday, Bristol Royal Hospital for Children;

Katherine Thompson, Chelsea and Westminster NHS Foundation Trust;

Michael Barrett, Children's Health Ireland at Crumlin;

Sheena Durnin, Children's Health Ireland at Tallaght;

Patrick Fitzpatrick, Children's Health Ireland at Temple Street;

Emma Fauteux, Cork University Hospital;

Hannah Walsh, Derriford Hospital, Plymouth;

Darren Ranasinghe, Epsom General Hospital;

Slyvester Gomes, Evelina London Children's Hospital;

Patrick Aldridge, Frimley Park Hospital;

Mark Anderson, Great North Children's Hospital, Newcastle Upon Tyne;

Phil Peacock, Great Western Hospital, Swindon;

Simon Richardson, Hull Royal Infirmary;

David Hartin, Ipswich Hospital;

Arshid Murad, James Cook University Hospital, Middlesbrough;

Nicholas Richens, John Radcliffe Hospital, Oxford;

Rachael Mitchell, King's College Hospital London;

Atif Latif, Kingston Hospital NHS Foundation Trust;

Alice Downes, Leeds General Infirmary;

Shane Fitzgerald, Leicester Royal Infirmary;

Claire Kirby, Newham Hospital, London;

Edward Snelson, Norfolk & Norwich University Hospitals;

Neha Jain, North Middlesex Hospital;

Pete Figg, Northern Devon Healthcare NHS Trust;

Lee Tubman, Northumbria Healthcare NHS Foundation Trust;

Paul Tanto, Northwick Park Hospital;

Christopher Gough, Nottingham University Hospitals NHS Trust;

Sharryn Gardner, Ormskirk & District General Hospital;

Alan Charters, Queen Alexandra Hospital, Portsmouth;

Gareth Patton, Royal Aberdeen Children's Hospital;

Michaela Lazner, Royal Alexandra Children's Hospital, Brighton;

Tom Waterfield, Royal Belfast Hospital for Sick Children;

Manish Thakker, Royal Berkshire NHS Foundation Trust;

Graham Johnson, Royal Derby Hospital;

Hannah Stewart, Royal Devon and Exeter Hospital;

Shye Wei Wong, Royal Free Hospital, London;

Jen Browning, Royal Hospital for Children & Young People, Edinburgh;

Steven Foster, Royal Hospital for Children, Glasgow;

David Kung, Royal Manchester Children's Hospital;

Kirsty Challen, Royal Preston Hospital;

Lorna Bagshaw, Royal Wolverhampton NHS Trust;

Stephen Davies, Salisbury NHS Foundation Trust;

Adrian Marsh, Shrewsbury & Telford NHS Trust;

Esther Wilson, Somerset Foundation Trust Musgrove Hospital;

Niall Mullen, South Tyneside & Sunderland NHS Foundation Trust;

Alasdair Moffat, Southampton Children's Hospital;

Ellie Day, Southmead Hospital, North Bristol Trust;

Heather Jarman, St George's University Hospitals NHS Foundation Trust;

Vanessa Merrick, St Mary's Hospital, Imperial College Healthcare NHS Trust;

Greg Cranston, The Grange Hospital, Newport;

Emre Basatemur, The Royal London Hospital;

Holly Brooker, Torbay and South Devon NHS Foundation Trust;

Tulsi Patel, University College London Hospital;

James Foley, University Hospital Galway;

Marylyn Emeda, University Hospital Lewisham;

George Simpson, University Hospital of North Tees;

Michael Fox, University Hospital of Wales, Cardiff;

Tadgh Moriarty, University Hospital Waterford;

Katherine Priddis, Watford General Hospital;

Rachel Shute, West Suffolk; Davin Amin, Wexham Park Hospital;

Amutha Anpananthar, Whipps Cross Hospital, London;

Alex Brown, Barnet Hospital;

Catherine Williams, Bolton NHS Foundation Trust;
